# Sex-specific effects of inbreeding on body colouration and physiological colour change in the cichlid fish *Pelvicachromis taeniatus*

**DOI:** 10.1186/s12862-022-02074-x

**Published:** 2022-10-31

**Authors:** Simon Vitt, Christina E. Bakowski, Timo Thünken

**Affiliations:** Institute of Evolutionary Biology and Ecology, University of Bonn, An der Immenburg 1, 53121 Bonn, Germany

**Keywords:** Sexual ornamentation, Stress response, Inbreeding, Colour variation, Dynamic colour expression

## Abstract

**Background:**

Colour expression is highly variable in animals. In fishes, rapid colour change, i.e. physiological colour change, can be observed in multiple contexts, e.g. in camouflage or communication, and is affected by various factors, such as stress. *Pelvicachromis taeniatus* is a cichlid fish from West Africa with sexual dichromatism and both sexes being brightly coloured and flexible in ornament expression. In the present study, inbred and outbred *P. taeniatus* were photographed before and after a stress situation to investigate the stress response regarding colour expression in both sexes.

**Results:**

The chromaticity and the colour patch size (relative coloured area at the abdomen) were determined at both timepoints and the changes were analysed. Additionally, the coefficients of variation within family groups for the chromaticity (CV_chromaticity_) and colour patch size (CV_area_) were calculated. Chromaticity as well as the extent of colouration increased significantly following handling stress. The change in chromaticity was not significantly different between in- and outbred individuals in females and males. Inbred males showed more intense yellow colouration than outbred males. Independent from inbreeding, the CV_chromaticity_ decreased following the handling stress. The change in CV_area_ of females and males differed between in- and outbred individuals. In females, the decrease was significantly stronger in inbred individuals and in males the decrease was stronger in the outbred group.

**Conclusion:**

The results show that short-term stress can increase colouration, potentially advertising individual’s stress tolerance. Furthermore, this study shows positive inbreeding effects on a sexually selected trait.

## Background

Colouration has a broad range of functions and is involved, e.g. in camouflage [1], thermoregulation [2], photoprotection [3], mimicry [4] and aposematism [5]. Another major aspect is communication within species, in both inter- and intrasexual interactions, as well as between species [6]. Variation of colour signals has been observed in terms of changing environmental conditions, such as ambient light [7] or turbidity [8]. Another major driver of colour variation is the individual quality, which can cause a condition-dependent expression of colour signals [9, 10]. Direct responses in changing colouration are mainly described in fishes regarding camouflage / antipredation or UV-protection [11].

Stress is also a factor which can induce changes in colouration. Effects of long-term stress, e.g. environmental stress, have been observed frequently in many species [12, 13], including fishes [14, 15]. In convict cichlids, *Amatitlania nigrofasciata*, guanine-based structural colouration is affected by ultraviolet-B-induced oxidative stress [14]. In contrast, immediate responses to short-termed stress in form of changes in colouration have been less explored. Carotenoid-based colouration is also described to be prone to stress. Carotenoids are involved in many physiological processes [16] and, thus, represent a potential key resource for allocation trade-offs between physiological processes, e.g. expression of colour signals and oxidative stress defence [17]. Stress, e.g. oxidative stress caused by immune challenges, is often followed by re-allocation of carotenoids and a reduced carotenoid-based colouration (reviewed in [18]). Skin darkening, mainly mediated by α-melanocyte-stimulating hormone (αMSH), is also often mediated by stress, which was described for red porgy, *Pagrus pagrus*, with high densities causing darker skin [19]. Similar effects were observed in Arctic charr, *Salvelinus alpinus*, as a consequence of social subordination [20]. In Nile tilapia, *Oreochromis niloticus*, fish confinement or air exposure is followed by eye darkening [21]. Whereas stress-related reduced colouration and brightness are described frequently (see above), intensified expression of colour ornaments following stress seems to be a rare phenomenon in animals. Nevertheless, positive correlations between glucocorticoids and carotenoid-based colouration have been described in lizards and birds (reviewed in [18]). In Arctic charr, a positive connection between carotenoid skin pigmentation and stress responsiveness, i.e. confinement stress, was described [22].

The dynamic and context-dependent expression of colour ornaments is one of the fundamental processes during visual communication. Integumentary colouration in lower vertebrates, including fishes, is based on pigmented skin cells (pigment-based colouration), so called chromatophores, which can be subdivided into cells containing pigments that absorb specific wavelengths: melanophores (black), xantophores (yellow), erythrophores (red), and cyanophores (blue), as well as structures that reflect and scatter light by reflecting crystals (structural colouration): iridophores and leucophores [23]. In fishes, many chromatophores were described to possess cellular motility, which in addition allows changes in integumentary colouration regarding both the gradation or variety of colour (hue) and patterns [24]. Rapid changes in colouration are caused by intracellular transport of pigment organelles within the chromatophores, or shifts in angles of crystals inside iridophores, and described as physiological colour change [23]. The regulation of rapid changes in colouration is based either on hormones or neuronal regulation (reviewed in [23]). Glucocorticoid hormones, which are released during stress response, are correlated with colouration in lizards [25] and fishes [22].

Inbreeding is defined as mating among genetically related individuals and results in higher homozygosity and reduced genetic variability. This can lead to inbreeding depression, i.e. the reduced fitness of inbred offspring compared to outbred offspring. An increased probability of deleterious recessive alleles being expressed [26] can result in negative effects on growth, morphology and survival (reviewed in [27]). The strength of inbreeding depression is variable and depending on environmental conditions. Thus, negative inbreeding effects may only appear under stressful conditions [28]. Furthermore, inbreeding effects may depend on the inbreeding history of a population [29] and the strength of inbreeding depression may decrease after continuous inbreeding because recessive deleterious alleles are purged from a population or may increase because negative effects accumulate [27]. Because of inbreeding depression, some species avoid inbreeding [30]. Female guppies, *Poecilia reticulata*, produce more offspring following matings with less-related males than with more closely related ones and were more likely to have mated with less-related males [31]. Furthermore, polyandrous mating behaviour, i.e. increased matings, of female guppies following matings with brothers is described as postcopulatory inbreeding avoidance mechanism and may minimise inbreeding through a dilution effect [32, 33]. To note, the generality of inbreeding avoidance has been questioned and recently it was settled that this phenomenon is rare even in animals [34]. However, inbreeding can have positive effects and increase the inclusive fitness [35–37]. It is hypothesized that local adaptations may be balanced against inbreeding cost [38] which may result in absent inbreeding avoidance or, on the contrary, inbreeding preferences.

Despite comprehensive literature on inbreeding depression, knowledge on the effect of inbreeding on colour expression is scarce. Notably, in male guppies , sexual body colouration seems to be impaired following inbreeding [29, 39]. However, other studies could not show inbreeding effects on colouration in other populations of guppies [40] or in three-spined sticklebacks, *Gasterosteus aculeatus* [41].

In this study, the West African cichlid *Pelvicachromis taeniatus* was used to examine immediate changes in colouration following short-termed handling stress, which is known to increase cortisol levels in fishes [42]. This monogamous cichlid fish [43] with mutual mate choice and biparental brood care [37] is a well-established model organism, which inhabits riverine systems in Cameroon, Nigeria and Benin. *P. taeniatus* shows active inbreeding, i.e. a preference for closely related conspecifics during mate choice [37, 44, 45] and no sign of inbreeding depression has been discovered so far regarding juvenile growth and survival [37], egg and sperm traits ([46, 47], respectively). On the contrary, positive effects of inbreeding could even be shown in this species in form of improved cooperation during parental care in related pairs [37]. Genetic analyses supported the hypothesis of active inbreeding by showing that the natural Moliwe population is highly inbred [48]. This species shows a high degree of sexual dimorphism and dichromatism with both sexes being brightly coloured [49, 50]. Males show a yellow colouration on their ventral side, whereas females develop a purple ventral belly colouration. The chromaticity and extent of these coloured areas play an important role during courtship, mate choice as well as intra-sexual competition [49–52] and are the main subject of this study.

The present study examined the impact of short-termed handling stress on the expression and variation of body colouration in third generation in- and outbred *P. taeniatus*. Stress can cause resource allocation trade-offs, e.g. of carotenoids, and thus reduce colouration [17]. Consequently, a reduced colour expression as response to a handling procedure can be expected. Alternatively, short term stress could increase colour expression in order to visually signal alertness. As dynamic trait, the intensity of body coloration is usually only maximally expressed during interactions with conspecifics in *P. taeniatus*, e.g. during courtship or intra-sexual contests.

Initial colouration as well as the response to stress may be different between inbred and outbred individuals. Inbreeding increases genetic homozygosity at individual level, which increases the probability that recessive deleterious alleles are expressed. In this case, we expect less intense colour expression and less flexible responses of inbred individuals compared to outbred individuals. On the other hand, inbreeding could reduce the recombination load and maintain favourable parental gene combinations [53], potentially leading to higher colour values in inbred offspring. Furthermore, inbreeding decreases within-family genetic variability. Accordingly, this could result in reduced variation of colour expression within inbred families compared to outbred families.

## Results

**Fig. 1:**
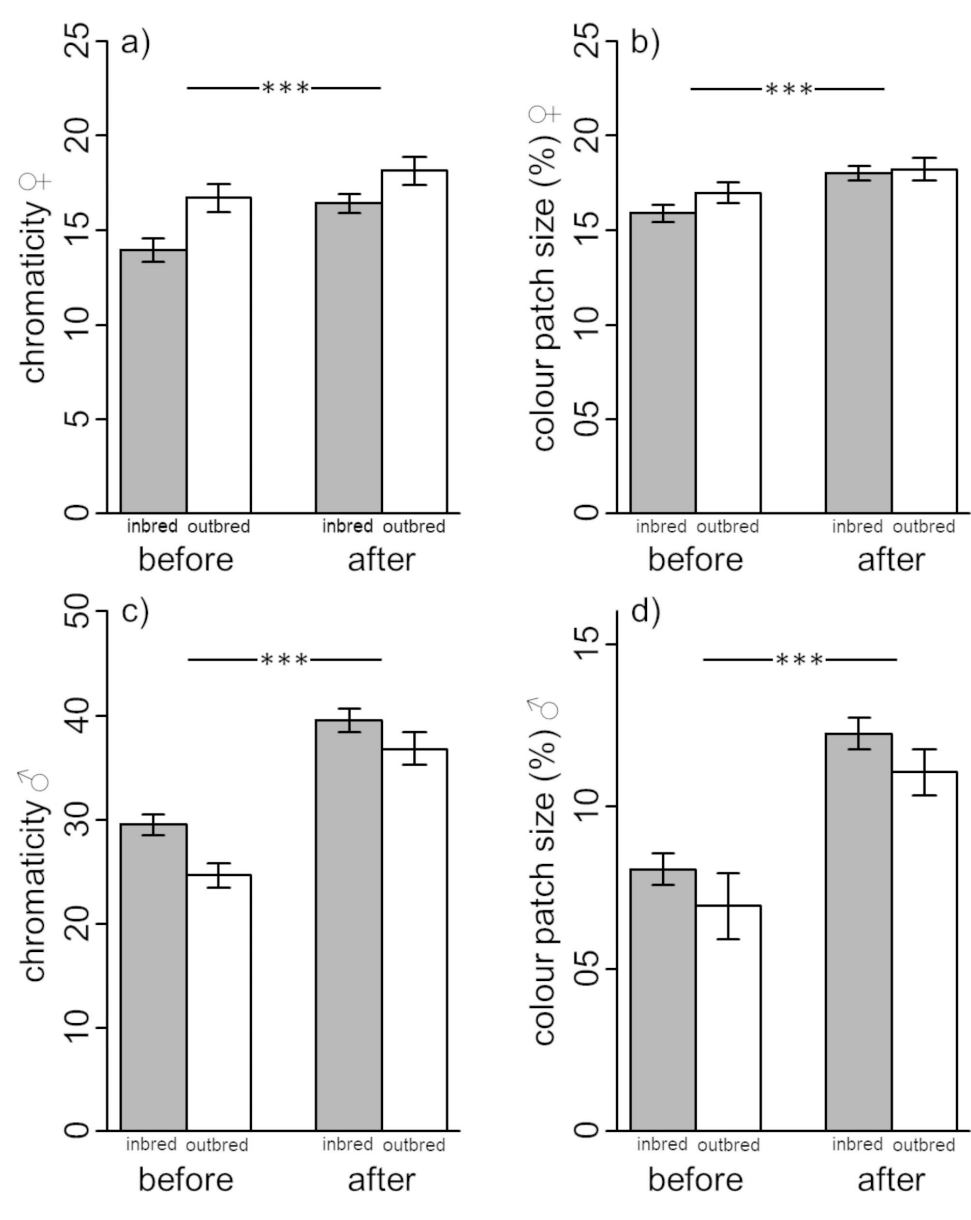
Shown are the chromaticity and colour patch size for the measures before and after stress exposure in females (**a**, **b**) and males (**c**, **d**). Given are means and standard errors for inbred (grey) and outbred (white) individuals. ***: p < 0.001

All measured colour variables, i.e. chromaticity, colour patch size and coefficients of variation significantly changed after the handling stress in both sexes of *P. taeniatus*. In detail, chromaticity and colour patch size increased between both timepoints in females and males but there was no significant effect of inbreeding (i.e. non-significant interaction terms) on the changes (Table1; Fig.1).

Inbred males were generally more intensively coloured than outbred males. Inbred and outbred males did not significantly differ in the extent of the coloured area (Table1; Fig.1). Inbred and outbred females did not significantly differ in both colour values (Table1; Fig.1).

**Fig. 2:**
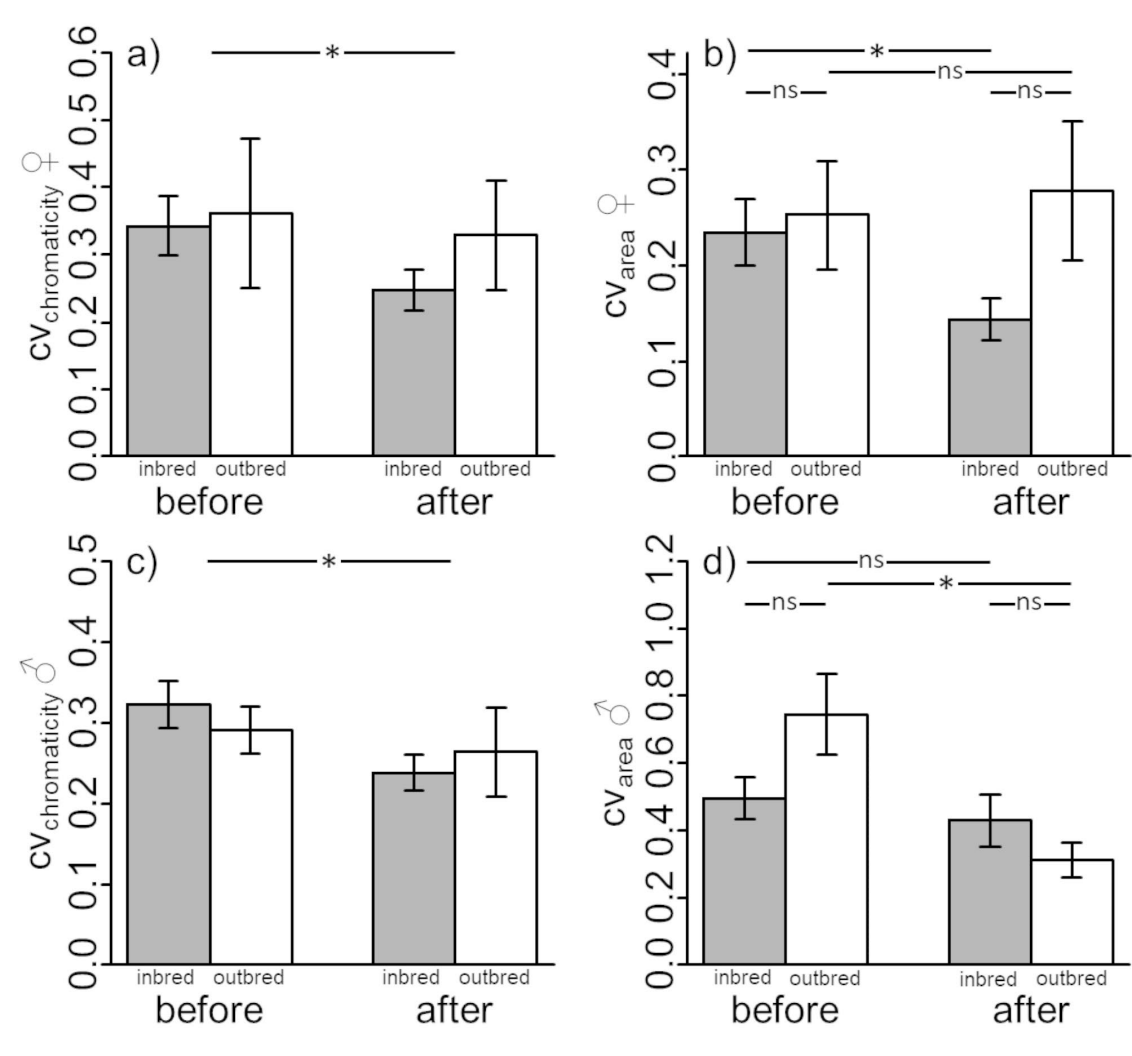
Shown are the coefficients of variation for the chromaticity and for the colour patch size for the measures before and after stress exposure in females (**a**, **b**) and males (**c**, **d**). Significant interaction terms (timepoint x inbreeding/outbreeding) were analysed using post-hoc test to calculate contrasts among the CV_area_ of inbred and outbred females and males between timepoints as changes differed between groups. Given are means and standard errors for inbred (grey) and outbred (white) individuals. ns: p > 0.05; *: p < 0.05;

Independent from in- or outbreeding, the CV_chromaticity_ decreased significantly in both sexes (Table2; Fig.2). The change in CV_area_ was significantly different between in- and outbred females and males (significant interaction term; Table2). Bonferroni adjusted post-hoc tests revealed a significant decrease of the CV_area_ between measurements in inbred females (Table2; Fig.2) and in outbred males. Post-hoc tests regarding the CV_area_ did neither show significant differences between inbred and outbred individuals of both sexes within measurement 1 nor within measurement 2 (Table2; Fig.2).

**Table 1:** All linear mixed-effects models calculated for chromaticity and colour patch size for both sexes. As random factors, families of father and mother and the ID of each fish were included in each linear model. Significant results are printed in bold (p < 0.05)

sex	dependent variable	R2m	R2c	explanatory variable	estimate	F	p
females	chromaticity	0.047	0.770	timepoint x inbreeding	-1.062	2.685	0.103
				inbreeding	2.891	1.128	0.326
				**timepoint**	**2.474**	**43.249**	**< 0.001**
	colour patch size	0.149	0.801	timepoint x inbreeding	-0.004	0.383	0.544
				inbreeding	0.007	0.009	0.924
				**timepoint**	**0.017**	**25.149**	**< 0.001**
males	chromaticity	0.285	0.756	timepoint x inbreeding	2.240	2.274	0.134
				**inbreeding**	**-8.782**	**5.408**	**0.034**
				**timepoint**	**9.977**	**235.420**	**< 0.001**
	colour patch size	0.236	0.772	timepoint x inbreeding	0.007	0.729	0.395
				inbreeding	-0.029	1.863	0.199
				**timepoint**	**0.048**	**197.337**	**< 0.001**

**Table 2:** All linear mixed-effects models calculated for the coefficients of variation (CV) regarding chromaticity and colour patch size for both sexes. As random factors, families of father and mothers and the ID of each fish were included in each linear model. Significant interaction terms were further analysed using post-hoc test with the Bonferroni correction method (t1 = before, t2 = after). Significant results are printed in bold (p < 0.05)

sex	dependent variable	R2m	R2c	explanatory variable	estimate	t	F	p
females	CV chromaticity	0.062	0.796	timepoint x inbreeding	0.062		1.047	0.321
				inbreeding	-0.036		0.350	0.570
				**timepoint**	**-0.095**		**5.021**	**0.039**
	CV colour patch size	0.165	0.821	**timepoint x inbreeding**	**0.927**		**10.146**	**0.005**
				inbreeding	-0.918		1.269	0.276
				**timepoint**	**-0.830**		**6.135**	**0.024**
	post-hoc tests			**t1 inbreeding vs. t2 inbreeding**	**0.830**	**4.175**		**0.003**
				t1 outbreeding vs. t2 outbreeding	-0.097	-0.408		> 0.999
				t1 inbreeding vs. t1 outbreeding	-0.010	-0.019		> 0.999
				t2 inbreeding vs. t2 outbreeding	-0.937	-1.852		0.512
males	CV chromaticity	0.156	0.300	timepoint x inbreeding	0.411		0.528	0.477
				inbreeding	-0.697		0.058	0.812
				**timepoint**	**-0.899**		**6.990**	**0.017**
	CV colour patch size	0.304	0.313	timepoint x inbreeding	-0.364		5.545	**0.028**
				inbreeding	0.612		0.479	0.503
				**timepoint**	**-0.068**		**7.236**	**0.013**
	post-hoc tests			t1 inbreeding vs. t2 inbreeding	0.068	0.634		> 0.999
				**t1 outbreeding vs. t2 outbreeding**	**0.432**	**3.496**		**0.016**
				t1 inbreeding vs. t1 outbreeding	-0.248	-1.943		0.364
				t2 inbreeding vs. t2 outbreeding	0.116	0.948		> 0.999

## Discussion

Inbred males showed more intense yellow colouration than outbred males. In females, there was no significant difference in colouration between in- and outbred individuals. In both sexes, the chromaticity and the colour patch size significantly increased between measurements as a response to stress by handling. However, no significant difference was observed regarding the change in chromaticity between in- and outbred individuals. The CV_chromaticity_ decreased following stress exposure while the changes in CV_area_ differed between sexes and between in- and outbred individuals.

In fishes, reduced expression of colour ornaments was observed frequently in both, pigment-based and structural colouration. For example, two species of Malawi cichlids, *Melanochromis auratus* and *Metriaclima zebra*, showed reduced pigment-based colouration following stress by handling, anaesthesia or enhanced ambient light intensity [54]. Convict cichlids expressed reduced guanine-based structural colouration following stress by ultraviolet-B radiation [15]. In contrast, stress-induced increase in colouration seems to be a rare phenomenon. Nevertheless, glucocorticoids, which are released during stress responses, were shown to promote colouration in the common lizard, *Lacerta vivipara*, with elevated blood corticosterone levels being associated with increased redness of the lizard’s belly [25]. In Arctic charr, carotenoid pigmentation seems to be linked to stress-coping style as a connection was observed between carotenoid skin pigmentation and stress responsiveness [22]. In the present study, a physiological colour change occurred as an immediate response to stress and resulted in enhanced colouration. Nevertheless, because we could not quantify potential changes in colouration between catching the experimental fish and taking the first photograph, individuals might have already expressed a colour change preceding the first measurement and may have recovered until the second photo. However, photos were taken quickly (within 30s) and fish were handled and afterwards kept individually in small 1-L plastic boxes with no hiding place between both photographs. Thus, a recovery and potential return to the baseline colouration (before catching) between both photographs is very unlikely. In addition, no evident change in colouration could visibly be detected between catching and taking the first photograph. Measuring blood cortisol levels, which are positively correlated with stress in fishes [55], could be used to quantify stress by handling but would also be accompanied by an invasive procedure which was avoided in the present study.

Enhancing colouration following stress is discussed by KM Sefc, AC Brown and ED Clotfelter [18] as a form of advertising stress tolerance (glucocorticoid tolerance). Accordingly, a relationship between hormone levels and carotenoid colouration was observed in the common lizard [56]. Advertising stress tolerance can be beneficial in the mate choice context as it may represent a quality-related trait in terms of the ability to cope with changing environments or predatory threats. Additionally, displaying stress tolerance may improve competitiveness in an intrasexual context, e.g. during competition for food resources, territories or mating partners. In *P. taeniatus*, adult males and females face strong intrasexual competition [45, 57, 58]. Here, a rapid-onset unpredictable stress event caused an acute stress response in form of intensified colouration which may be advantageous during a sudden attack by a conspecific as it may visually signal alertness and defensiveness.

Inbred and outbred fish did not differ in the change in colouration which is in line with previous studies that also did not find significant inbreeding depression [37, 46, 47, 59]. In contrast, in the present study, inbred males *P. taeniatus* showed a significantly higher chromaticity. In *P. taeniatus*, females prefer males that show a bright yellow belly colouration over less intense coloured males [50]. Thus, individuals originating from inbred groups might be favoured during mate choice when competing with outbred individuals which may ultimately lead to fitness benefits. Beside potential genetic benefits resulting from inbreeding due to purging deleterious alleles and the maintenance of co-adapted gene complexes in inbred individuals, inbreeding may also affect the social behaviour in groups. Colour expression in *P. taeniatus* may be associated with social behaviour as it plays an important role in intra-specific communication [51, 52]. Inbreeding increases the genetic relatedness between siblings, which may facilitate cooperation according to the inclusive fitness theory developed by WD Hamilton [60], predicting that the genetic relatedness is important for the evolution of cooperation and group living [61]. Accordingly, increased relatedness in inbred groups might have facilitated cooperation and / or reduced social stress within the group. In *P. taeniatus*, relatedness of mating partners promotes cooperation during parental care [37], which could potentially affect other social interactions as well. Social stress can impair colouration, which has been observed, e.g. in Arctic charr with darkening skin as social subordination [20]. In our case, enhanced cooperation and reduced social stress in inbred groups might have promoted resource allocation directed towards colouration. Alternatively, increased kin competition within inbred groups might lead to higher aggression [62], which is associated with more intense yellow colouration in male *P. taeniatus* [52]. In *P. taeniatus*, males compete for access to breeding sites and females. In addition, the defence of territories is mediated by aggressive behaviour of males against rivals which makes males the more competitive sex in this species. Related competitors elicited higher aggression in territorial males than unrelated competitors [57]. Explorative behaviour [63] and increased risk of kin competition also increases juvenile shoaling behaviour [64]. Absent effect of inbreeding on female *P. taeniatus* may be explained by the generally higher sociality in females as they form loose groups when searching for territory holding males.

Short-termed stress and the following increase in colouration reduced variation in colour intensity within groups of full siblings in both sexes. Comparably higher initial variation can have various causes. Colouration in fishes is affected by multiple factors including diet, health status and social interactions (reviewed in [18]). Colouration at the first measurement most likely represent the variation within family groups, which is dependent on dominance relationships. Especially in males, only the dominant individuals are able to express pronounced colouration. Our results suggest that stress triggers the maximal possible colouration in both dominant and subdominant males levelling the initial difference between males. Concerning change in CV_area_, only outbred males showed a significant reduction, while variation in inbred males remained unchanged at a relatively low level. More interestingly, in outbred females, in contrast to outbred males, CV_area_ stays similar between measurements. This is probably due to the fact that initial variation in males was much higher than in females (0.7 vs. 0.25). In contrast to outbred females, variation in colour patch size of inbred females was reduced in the second measurement, likely due to reduced stress tolerance in inbred females.

## Conclusion

In conclusion, this study shows that short-termed stress can enhance sexually selected colour expression in *P. taeniatus*. Moreover, we showed that inbreeding affects the colour expression in males. This study also reveals sex-specific differences in the change of colour variation between inbred and outbred *P. taeniatus*. Whether the colour-enhancing effect of short-termed stress still occurs under more challenging environmental conditions, e.g. including predation, pathogens or restricted resources, remains opened. However, the ability of immediate colour responses is an important trait in fishes which may enhance individual’s survival, e.g. by predator intimidation, or direct fitness by signalling physical quality during mate choice.

## Methods

### Experimental animals

The experimental animals used in this study were reproductively active males and females from the third generation of laboratory bred *P. taeniatus* descending from wild fish of the Moliwe River (near Limbe, Cameroon 04°040N/09°160E) that were bred under standardized conditions at the Institute of Evolutionary Biology and Ecology of the University of Bonn (see [37]). Inbred individuals were bred by continuous full sibling matings across generations and outbred individuals by matings between unrelated individuals. After spawning, eggs were removed from the parents and transferred to 1L plastic boxes equipped with an airstone. At the age of one month, juveniles were transferred to tanks measuring 30 × 20 × 20cm (length × width × height; l x w x h) and at the age of three months to tanks measuring 50 × 30 × 30cm (l × w × h). All tanks were equipped with sand as substrate, Java moss, (*Vesicularia dubyana*) as shelter and Tetra Brillant Filters (Tetra Co. Ltd, Japan). During the first two months, fish were fed with freshly hatched *Artemia* nauplii six days a week *in excess*. From the third month on, all fish were fed five days a week *in excess* with a mixture of defrosted *Artemia sp.*, red, (*Chironomus sp.*), black (*Culex sp.*) and white (*Chaoborus sp.*) mosquito larvae. All tanks were visually separated from each other by opaque plastic sheets. The water temperature was kept at 25 ± 2°C and the light-dark cycle was set to 12:12h light:dark.

All experimental fish were kept in mixed-sex groups and sexes were determined visually, based on the sex-specific colouration (see above). In total, 183 inbred (96 females, 87 males; 11 families) and 97 outbred fish (57 females, 40 males; 7 families) were used. Inbred fish had an age of on average 855 ± 2 days (mean ± standard error, SE) and outbred fish of 851 ± 2 days (mean ± SE) when being used in the experiment. For all experimental fish, standard length (length from the tip of the mouth to the base of the caudal fin) was measured to the nearest millimetre using scale paper. Inbred females had a standard length of 3.96 ± 0.02cm and outbred females of 4.01 ± 0.03 (mean ± SE) and inbred males of 6.06 ± 0.07cm and outbred males of 6.05 ± 0.10 (mean ± SE). Neither age nor standard length differed significantly between inbred and outbred females (age: Wilcoxon-Test, W = 3140.5, p = 0.127; standard length: Wilcoxon-Test, W = 2447, p = 0.273) and males (age: Wilcoxon-Test, W = 1875, p = 0.485; standard length: Wilcoxon-Test, W = 1774.5, p = 0.860).

### Photographs

Photography has advantages over spectrophotometry to quantify colouration in this species. Photographs can be taken quickly with individuals being underwater to minimize stress, which is important as *P. taeniatus* rapidly changes its colour under stress. Additionally, spectrophotometric data and CIELab data from photographs are highly correlated for this species [52, 65].

All photographs were taken using a standardized set-up as described in L John, IP Rick, S Vitt and T Thünken [52]. A photo-box made of Plexiglas (9.5 × 15 × 7cm, l × w × h) was filled with temperate water (25 ± 2°C) and placed at a distance of 84cm in front of a macro objective (AF-S Micro Nikkor 105mm 1:28G) attached to a digital single lens reflex camera (Nikon D5000). The photo-box was illuminated by two light sources (16W LED lamps, Toshiba LDRC1665WE7EUD, 32°, 6500K) at a distance of 36cm and an angle of 35° to the photo-box. A colour standard (Munsell colour standard chip) and a size reference (a piece of graph paper) were attached to the photo-box apparatus.

Two photographs were taken from each experimental animal. The first photograph was taken immediately after the individual was caught out of its group tank and the second photograph was taken after the stress exposure in form of the handling procedure for measuring standard length and body mass. For taking the first photograph, experimental fish were haphazardly chosen and individually caught in their holding tanks using a dip net. Individuals were transferred to the photo set-up, which was build up close to the holding tank, using a 1-L plastic box. They were carefully placed in the photo-box by hand and held in place (in the middle of the box) using a plastic pipette. Photos were taken using a remote-trigger and saved in RAW-format in order to preserve maximum image information. After taking the first photograph, the standard length (to the nearest mm) and body mass (to the nearest mg) of each individual were determined and fish were placed back in the 1-L plastic box for two minutes before the second photograph was taken as described above.

### Colour analyses

Colour analyses were conducted in the same way for both measures of each individual. Based on the digital photographs, the chromaticity of the body colouration was quantified using the program Adobe Photoshop CS4 Extended (Version 11.0.2). First, during the import of RAW-photos, light temperature was adjusted to the light temperature of the light sources (6500K). Subsequently, the white standard on the photograph was used to adjust the white balance. The colour space of the image was set to the standardized and device independent three-dimensional CIELab colour space, with the L- (lightness), a- and b-value (colour) on three axes [66]. CIELab values are an established measure of animal colouration and often used in studies examining ornamental traits in fishes, including *P. taeniatus* (e.g. [49, 52, 65]).

To measure the chromaticity of the ventral colouration (yellow colouration for males and purple colouration for females), the colour-sampler tool in Adobe Photoshop was used to take 12 samples for males and 16 samples for females (5 pixels x 5pixels each), evenly spread over the coloured area at the belly (for details, see [52]). The chromaticity of each sample was calculated from the a- and b-values using the following formula according to AR Robertson [67]:$chromaticity= \sqrt{{a}^{2}+{b}^{2}}$

The extent of the ventral colouration was determined using the program ImageJ (Version 1.53e). First, the lateral projection area, excluding the fins, was selected and measured using the polygon tool. Second, the coloured area was measured by selecting pixel of a specific colour using the ‘colour threshold’ and ‘analyse particles’ option. For females, the hue was set to 154–255, saturation to 5–255 and brightness to 66–255. For males, the hue was set to 0–64, saturation to 100–255 and brightness to 66–255 [52]. By dividing the coloured area by the lateral projection area, the relative colour patch size was calculated.

The coefficient of variation (CV) was calculated for the chromaticity (CV_chromaticity_) and colour patch size (CV_area_) for both measures, based on the values within family groups. The CV is a common measure for variation [68] and was used in studies on animal ornamentation [69]. Here, the CV_chromaticity_ CV_area_ describe the variation of colour expression within families.

### Statistical analysis

Statistical analysis was performed in R, version 4.1.1 [70]. Differences in standard lengths and ages within females and males, between in- and outbred individuals were tested using independent Wilcoxon-Tests. Colour variables and coefficients of variation were analysed by conducting linear mixed-effects models (LME) using the lme4-package [71]. LMEs included an interaction term consisting of the category variable ‘inbreeding/outbreeding’ and the timepoint of measurement (measure 1 / measure 2) as explanatory variables and the ID of each individual and families of mothers and fathers as random factors.

Post-hoc tests were conducted to calculate contrasts among the CV_area_ of inbred and outbred females and males between timepoints as changes differed between groups (significant interactions between ‘inbreeding/outbreeding’ and the timepoint of measurement). Post-hoc tests were conducted using the R package ‘emmeans‘ based on comparisons between estimated marginal means (EMMs) and corrected for multiple comparisons using the Bonferroni method [72].

P-values were calculated using the backward elimination procedure of the ‘step‘ function in the lmerTest package [73]. Significance values for the fixed effects were based on F-tests with Kenward-Roger approximation. Normal distributions of the residuals of all best-explaining models were tested using the ‘check_normality’ function of package performance [74]. Residuals of models including the colour patch size and the CV_area_ of females as well as the CV_chromaticity_ and the CV_area_ of males were not normal distributed. Thus, regarding variables were Box–Cox-transformed [75] to meet the assumptions of normality. Residuals of the best explaining models including the Box–Cox-transformed variable ‘relative coloured area’ of females was still not normal distributed. Therefore, averages were calculated for families and used for the LME and the normality of the residuals was confirmed. The marginal R^2^ (R^2^m) and the conditional R^2^ (R^2^c) were calculated in R using the MuMIn package [76].

## Data Availability

All data generated or analysed during the current study are included in this published article and its supplementary information files.
